# Description of new species *Macellicephaloides veronikae* sp. n. (Polynoidae, Annelida) from the Amundsen Sea, Southern Ocean

**DOI:** 10.1007/s00300-025-03414-5

**Published:** 2025-10-03

**Authors:** Lenka Neal, Helena Wiklund, Adrian G. Glover

**Affiliations:** 1 Department of Life Sciences, Natural History Museum, Cromwell Road, London, SW7 5BD UK; 2 Department of Marine Sciences, University of Gothenburg, Gothenburg, Sweden; 3 Gothenburg Global Biodiversity Centre, Gothenburg, Sweden

**Keywords:** Taxonomic novelty, Molecular phylogeny, 16S, 18S, Antarctic shelf, Deep sea

## Abstract

While the Southern Ocean represents a unique habitat, currently undergoing rapid environmental change, its biodiversity remains largely unknown, particularly at greater depths. Increased sampling efforts in the Amundsen Sea, a previously unexplored region of the Southern Ocean, combined with the use of an epibenthic sledge resulted in a large collection of mobile, scale-bearing worms from the family Polynoidae Kinberg, 1856. The greatest taxonomic novelty in the material collected from the Pine Island Bay, Amundsen Sea, was found within the exclusively deep-sea subfamily Macellicephalinae Hartmann-Schröder, 1971. Examination of this material has already led to formalization of six new species of *Macellicephala* (Neal et al. 2018). This study represents the continuation of such effort with formalization of *Macellicephaloides veronikae* sp. n. based on morphology and 16S and 18S molecular markers. In the phylogenetic analyses, the new species is sister taxon to *Macellicephaloides moustachu* from the abyssal equatorial Pacific Ocean, albeit based on very limited taxon sampling currently available. *Macellicephaloides veronikae* sp. n. shows the shallowest distribution (500–1000 m) of this genus recorded to date and may represent a case of polar emergence.

## Introduction

**Fig. 1:**
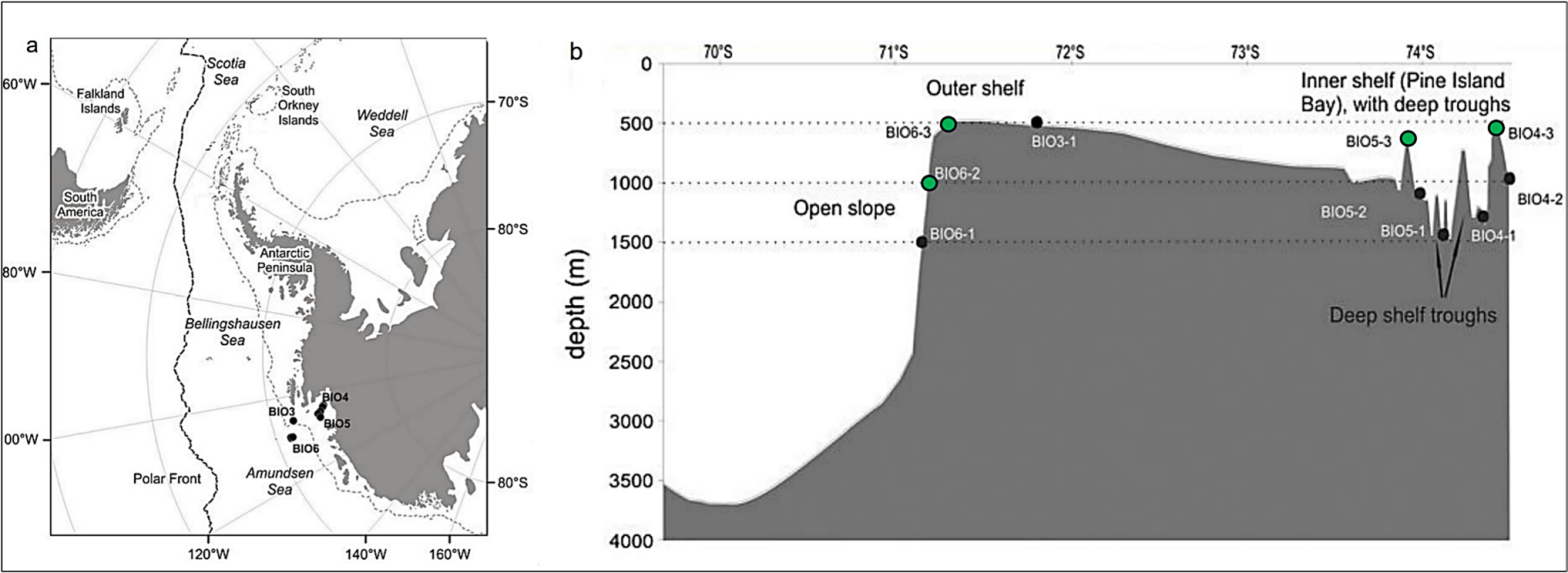
Map of sampling sites and background environmental information. **a** Map of sampling sites visited during the BIOPEARL II—JR179 cruise to the Amundsen Sea (black circles). **b** Depth cross-section of the shelf and slope in the Amundsen Sea, showing deep-trough sites on the inner shelf (Pine Island Bay) and outer shelf/slope. Green circle: samples with *Macellicephaloides veronikae* presented in this study. Black circle: overall sampling effort

The Southern Ocean, a unique and rapidly changing habitat, still has much of its biodiversity unexplored, especially at greater depths. The region’s deep-sea ecosystems remain poorly understood, highlighting the need for further research to uncover their biodiversity (e.g. Griffiths 2010). The Amundsen Sea (Fig. 1a) is a significant body of water situated in western Antarctica, bordered by the Bellingshausen Sea and the Ross Sea, undergoing rapid environmental change due to melting of the Pine Island Glacier, which flows into Pine Island Bay, the Amundsen Sea’s principal embayment (Reed et al. 2024). The past glacial actions have also created a complex bathymetry of the shelf by carving deep troughs up to 1500 m deep (Fig. 1b).

The first benthic samples collected from the Amundsen Sea in the Southern Ocean, as part of the BIOPEARL programme (Biodiversity, Phylogeny, Evolution and Adaptive Radiation of Life) (Linse et al. 2013), revealed significant taxonomic novelty in several macrofaunal groups, including annelid worms (e.g. Kaiser et al. 2009; Moreau et al. 2013; Pabis et al. 2015; Neal et al. 2018). These discoveries were particularly notable in deep-shelf and slope areas (500–1500 m), which had been underexplored in previous studies that focused on shallower depths (Griffiths 2010). Only the ANDEEP expeditions (ANtarctic benthic DEEP-sea biodiversity) had previously targeted deep-sea areas of the Southern Ocean, also finding high levels of novel biodiversity (e.g. Brandt et al. 2007; Schüller et al. 2009). The BIOPEARL programme employed a relatively new sampling device, the Brenke Epibenthic sledge (Brenke 2005), which helped capture highly mobile epibenthic fauna, including scale-bearing worms of family Polynoidae Kinberg, 1856, which had often been under-represented in earlier studies using coring devices (Neal et al. 2017).

Polynoidae, the largest family of scale-bearing annelids (Aphroditiformia), includes around 1000 species across approximately 160 genera and 8 subfamilies, making it one of the most diverse families among polychaetes (Read and Fauchald 2025). These errant worms were once thought to have a broad geographic and bathymetric distribution, although recent molecular studies have challenged this view (e. g. Bogantes et al. 2020; Serpetti et al. 2017). In the Southern Ocean, there are approximately 60 valid species of Polynoidae, which represents about 7.5% of the known polychaete fauna in the region estimated at around 800 species (Schüller and Ebbe 2014). Most polynoid diversity in the Southern Ocean has been observed in shallow waters (< 500 m), primarily in the subfamily Polynoinae Kinberg, 1856. However, some subfamilies are restricted to deep-sea habitats, including bathyal to hadal depths and chemosynthetic environments or shallow deep-sea analogues such as submarine caves, with the subfamily Macellicephalinae Hartmann-Schröder, 1971, being particularly prominent in such habitats.

Neal et al. (2018) already described in part the novelty found within the Antarctic Macellicephalinae by formalizing six new species of *Macellicephala*. Here, we continue the efforts of describing the polynoid fauna of the Southern Ocean with the formalization of new species in the genus *Macellicephaloides*. Genus *Macellicephaloides* Ushakov, 1955, was erected to accommodate three species discovered from the Kuril-Kamchatka Trench—*M. grandicirra* Uschakov, 1955, *M. verrucosa* Uschakov, 1955, and *M. vitiazi* Uschakov, 1955. Subsequent discovery of further four species from the trenches supported the hypothesis that the genus *Macellicephaloides* is adapted to hadal depths – *M. uschakovi* Levenstein, 1971, from the Kuril-Kamchatka Trench; *M. improvisa* Levenstein, 1982; and *M. villosa* Levenstein, 1982, from the Trench of Japan and *M. sandvichensis* Levenstein, 1975, from the South Sandwich Trench in the Atlantic sector of the Southern Ocean. Pettibone (1989a) described *M. alvini*, a bathyal representative, from the bacterial mats at hydrothermal vents in the Guaymas Basin, Gulf of California, at a depth of 2004 m. It took nearly three decades to describe the next species in this genus—*M. moustachu* Bonifácio and Menot, 2018, from the polymetallic nodule fields of the Clarion-Clipperton Zone in the abyssal equatorial Pacific. The discovery of *Macellicephaloides veronikae* sp. n., from the depths of ~ 500–1000 m in the Amundsen Sea, Southern Ocean, formalized here, is thus the shallowest record of this genus to date and may be an example of polar emergence (e.g. Brandt 1992; Strugnell et al. 2011), recently suggested for some, but not all Antarctic polynoids (Cowart et al. 2022).

## Material and methods

### Field sampling and laboratory analysis

Samples were collected during the BIOPEARL II expedition to the Amundsen Sea in austral summer of 2007/2008 onboard RRS James Clark Ross and organized by the British Antarctic Survey (see Linse et al. 2013 for details). Targeted depths were 500 m, 1000 m, and 1500 m horizons. The detailed description of epibenthic sledge (EBS) is given in Brenke (2005) and Glover et al. (2016). Briefly, the EBS consists of a 500-µm epi- (lower) and a supra- (upper) net, each with an opening of 100 cm width and 33 cm height. Both nets end up in cod ends with a mesh size of 300 µm. The EBS was hauled over the seabed at 1 knot for 10 min. The study areas, field methods, and treatment of polychaete samples on board and in the laboratory were described in detail in Neal et al. (2017), using protocols detailed in Glover et al. (2016).

#### SEM

Paratype NHMUK. 2018.246 was examined using SEM. The specimen was dehydrated in 100% alcohol, critically point dried, coated with gold-vanadium, and examined using Phillips XL30 SEM at the Imaging and Analysis Centre, Natural History Museum, London.

### Molecular analysis

In total, DNA was extracted from 15 specimens morphologically assigned to genus *Macellicephaloides* in Neal et al. (2017). Holotype NHMUK. 2018.915 was targeted in this study with GenBank accession numbers PV911683 for 16S and PV911684 for 18S. Other specimens were targeted by Brasier et al. (2016), with GenBank accession numbers KX867331- KX867344.

DNA was extracted from parapodia using DNeasy Blood and Tissue Kit (Qiagen) following the protocol supplied by the manufacturer, with final elution in 100 μl buffer. Two genes were amplified: the mt non-coding 16S and the nuclear (n) non-coding 18S gene. About 450 bp of 16S and 1800 bp of 18S were amplified using the following primers: Ann16SF and 16SbrH for 16S (Palumbi 1996; Sjölin et al. 2005), and 18SA, 18SB, 620 F, and 1324R for 18S (Cohen et al. 1998; Medlin et al. 1988; Nygren and Sundberg 2003).

PCR mixtures contained 1 μl of each primer (10 μM), 2 μl template DNA, and 21 μl Red Taq DNA Polymerase 1.1X MasterMix (VWR) in a mixture totalling 25 μl. The temperature profile was as follows: 96 °C for 240 s, followed by (94 °C for 30 s, 48 °C for 30 s, then 72 °C for 60 s)*35 cycles, followed by 72 °C for 480 s. PCR purification was performed using a Millipore Multiscreen 96-well PCR Purification System, and sequencing was performed on an ABI 3730XL DNA Analyser (Applied Biosystems) at the Natural History Museum Sequencing Facility, using the primers mentioned above.

Overlapping sequence fragments were merged into consensus sequences using Geneious Prime 2025.0.3 (https://www.geneious.com). Gene 18S was used for the phylogenetic analyses. The 18S sequences were aligned together with other Polynoidae sequences and with *Neoleanira tetragona* (Örsted 1845) from Sigalionidae as root, using MAFFT (Katoh et al. 2002) with default settings, provided as plug-in in Geneious. Maximum likelihood (ML) analysis was performed using IQTree 1.6.0 (Nguyen et al. 2015) where ModelFinder (Kalyaanamoorthy et al. 2017) selected the optimal model TIMe + I + G4 for 18S. ML was run with 1000 bootstrap replicates. Bayesian phylogenetic analyses (BAs) using the model GTR + I + G were conducted with MrBayes 3.2.6 (Ronquist et al. 2012). Analyses were run independently three times for 10,000,000 generations. Of these, 2,500 000 generations were discarded as burn-in. The tree files were interpreted with FigTree v1.4.2 (http://tree.bio.ed.ac.uk/software/figtree/) and edited using Sketch (https://www.sketch.com).

All sequences obtained in this study have been deposited in GenBank (http://www.ncbi.nlm.nih.gov/genbank), with sequences deposited by Brasier et al. (2016) already available. GenBank sequence accession numbers are summarized in Table 1. Type material was deposited in the Natural History Museum, London, UK (NHMUK).

**Table 1:** List of taxa included in the phylogenetic analysis, their GenBank accession numbers and their current placement within subfamilies of Polynoidae

Taxon name	Genbank acc. no	Family/subfamily
*Acholoe squamosa* (Delle Chiaje, 1827)	AY839567.1	Polynoinae
*Antarctinoe ferox* (Baird, 1865)	MG905039.1	Polynoinae
*Bathyedithia retierei* Bonifácio & Menot, 2018	MH233215.1	Macellicephalinae
*Bathyeliasona mariaae* Bonifácio & Menot, 2018	MH233204.1	Macellicephalinae
*Bathyfauvelia glacigena* Bonifácio & Menot, 2018	MH233236.1	Macellicephalinae
*Bathyfauvelia ignigena* Bonifácio & Menot, 2018	MH233246.1	Macellicephalinae
*Bathykurila guaymasensis* Pettibone, 1989a	DQ074765.1	Macellicephalinae
*Bathymoorea lucasi* Bonifácio & Menot, 2018	MH233224.1	Eulagiscinae
*Bathypolaria magnicirrata* (Neal et. al., 2012)	JX863895.1	Macellicephalinae
*Branchipolynoe pettiboneae* Miura & Hashimoto, 1991	KU507074.1	Macellicephalinae
*Bruunilla nealae* Bonifácio & Menot, 2018	MH233216.1	Macellicephalinae
*Bylgides elegans* (Théel, 1879)	JN852822.1	Polynoinae
*Cladopolynoe sandersi* Hiley et al., 2024	JN852821.1	Macellicephalinae
*Eulagisca gigantea* Monro, 1939	MG905040.1	Eulagiscinae
*Gastrolepidia clavigera* Schmarda, 1861	JN852825.1	Arctonoinae
*Gorgoniapolynoe caeciliae* (Fauvel, 1913)	KU738172.1	Polynoinae
*Gorgoniapolynoe corralophila* (Day, 1960)	KU738175.1	Polynoinae
*Halosydna brevisetosa* (Kinberg, 1856)	JN852827.1	Lepidonotinae
*Halosydnella australis* (Kinberg, 1856)	KY823449.1	Lepidonotinae
*Harmothoe imbricata* (Linnaeus, 1767)	AY340434.1	Polynoinae
*Hermenia verruculosa* Grube, 1856	JN852830.1	Lepidonotinae
*Hodor anduril* Bonifácio & Menot, 2018	MH233239.1	Macellicephalinae
*Hodor hodor* Bonifácio & Menot, 2018	MH233238.1	Macellicephalinae
*Hyperhalosydna striata* (Kinberg, 1856)	JN852831.1	Lepidastheniinae
*Intoshella dictyaulus* Sui et al., 2018	MG519807.1	Polynoinae
*Lepidonotus clava* (Montagu, 1808)	JN852833.1	Lepidonotinae
*Macellicephala brenesorum* Neal et al., 2018	MG905041.1	Macellicephalinae
*Macellicephala clarionensis* Bonifácio & Menot, 2018	MH233235.1	Macellicephalinae
*Macellicephala gloveri* Neal et al., 2018	MG905042.1	Macellicephalinae
*Macellicephala linseae* Neal et al., 2018	MG905043.1	Macellicephalinae
*Macellicephala monroi* Neal et al., 2018	MG905044.1	Macellicephalinae
*Macellicephala parvafauces* Bonifácio & Menot, 2018	MH233225.1	Macellicephalinae
*Macellicephala patersoni* Neal et al., 2018	MG905045.1	Macellicephalinae
*Macellicephala violacea* (Levinsen, 1886)	OP476757.1	Macellicephalinae
*Macellicephaloides alvini* Pettibone, 1989a	OP651045.1	Macellicephalinae
*Macellicephaloides moustachu* Bonifácio & Menot, 2018	MH233212.1	Macellicephalinae
***Macellicephaloides veronikae* sp. n**	**PV911683-84**	Macellicephalinae
*Malmgrenia mcintoshi* (Tebble & Chambers, 1982)	JN852834.1	Polynoinae
*Neoleanira tetragona* (Örsted, 1845)	AY839570.1	Sigalionidae
*Neopolynoe acanellae* (Verrill, 1882)	MN653050.1	Polynoinae
*Nu aakhu* Bonifácio & Menot, 2018	MH233209.1	Macellicephalinae
*Pelagomacellicephala iliffei* Pettibone, 1985	KY454411.1	Macellicephalinae
*Photinopolynoe elytropapillata* (Zhang et al., 2018)	MG799378.1	Macellicephalinae
*Polaruschakov lamellae* Bonifácio & Menot, 2018	MH233226.1	Macellicephalinae
*Polaruschakov omnesae* Bonifácio & Menot, 2018	MH233213.1	Macellicephalinae
*Polynoe scolopendrina* Savigny, 1822	JN852839.1	Polynoinae
*Robertianella synophthalma* McIntosh, 1885	MN653053.1	Polynoinae
*Themis undomarginata* (Zhang et al., 2018)	MG799379.1	Macellicephalinae
*Thormora jukesii* Baird, 1865	JN852840.1	Lepidonotinae

Bold values indicates outlined the new species

In total, 49 terminal taxa were used in the molecular phylogenetic analysis (Table 1). Polynoidae were represented with 28 taxa from the subfamily Macellicephalinae, 11 from Polynoinae, five from Lepidonotinae Willey, 1902, two from Eulagiscinae Pettibone, 1997, and one each from Arctonoinae Hanley, 1989, and Lepidastheniinae Pettibone, 1989a, b (in Pettibone 1989b).

## Results

### Phylogeny

**Fig. 2:**
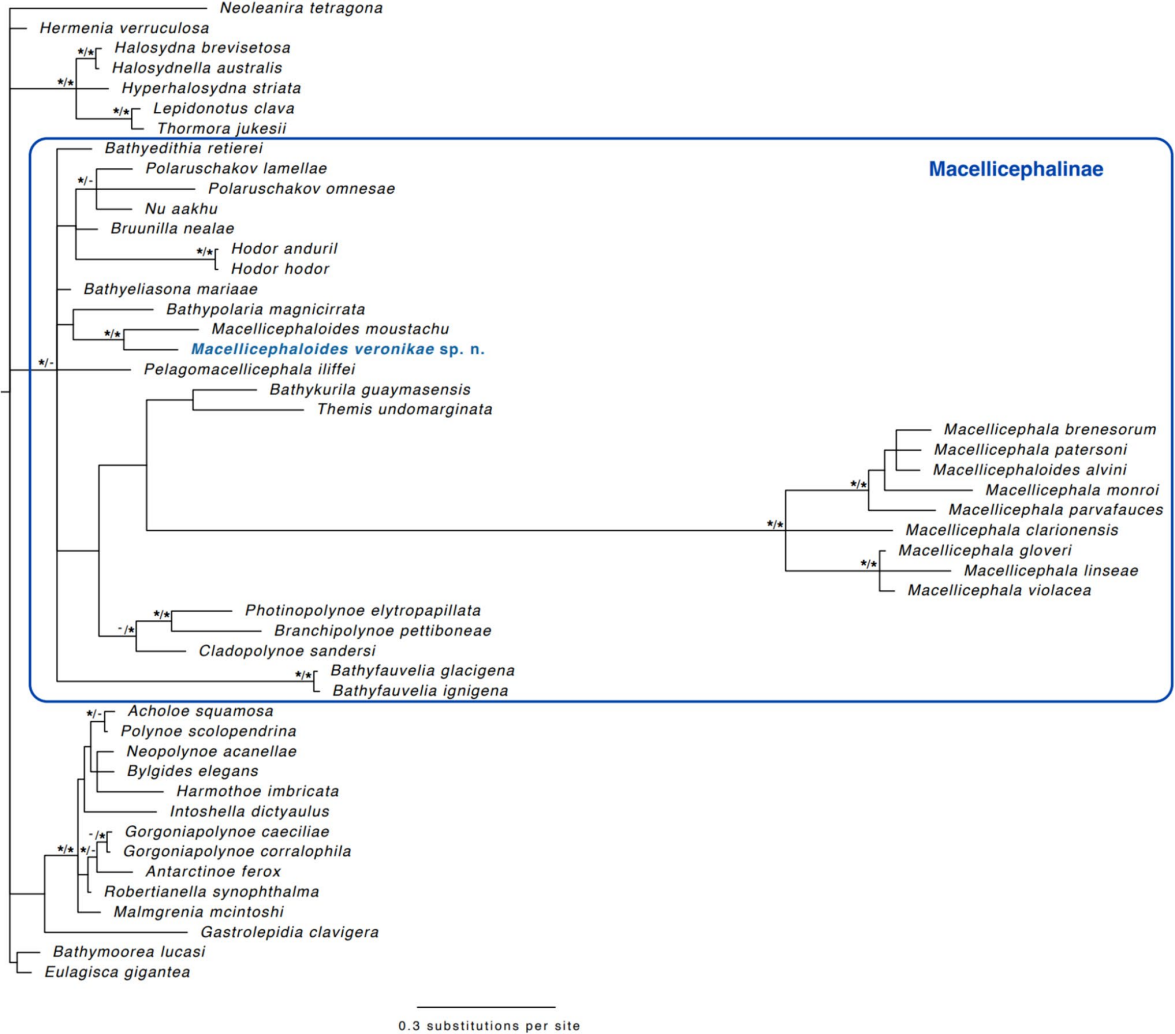
Majority rule consensus tree from the Bayesian Analyses (BA) using 18S, with 48 taxa from Polynoidae and *Neoleanira tetragona* (Sigalionidae) used as root. The bootstrap support values from the maximum likelihood (ML) analyses are added in to the Bayesian tree as BA/ML on the nodes. Support values at or above 0.95 for the BA and at or above 80 from the ML are shown in the tree as asterisks. Support values from both analyses are shown as */* while if support is low from one of the analyses it is shown as */- or -/*. No symbols on nodes show there was low support in both analyses

The phylogenetic analyses (Fig. 2) recovered *Macellicephaloides veronikae* sp. n. nested within the subfamily Macellicephalinae in agreement with Bonifácio and Menot (2019), as sister taxon to *Macellicephaloides moustachu* based on the currently available data. On GenBank, there are also sequences from a taxon labelled *Macellicephaloides alvini* Pettibone 1989a, b, which we included in our study. However, in our analyses, this taxon falls within the genus *Macellicephala* with strong support, which might be due to sequence contamination, or the sequenced specimen being misidentified.

### Systematics

Family: Polynoidae Kinberg, 1856

Subfamily: Macellicephalinae Hartmann-Schröder, 1971.

*Macellicephaloides* Uschakov, 1955

*Macellicephaloides veronikae* sp. n.

*Macellicephaloides* sp. B in Brasier et al. (2016), Neal et al. (2017)

Figures 3–6, 7j

**Fig. 3:**
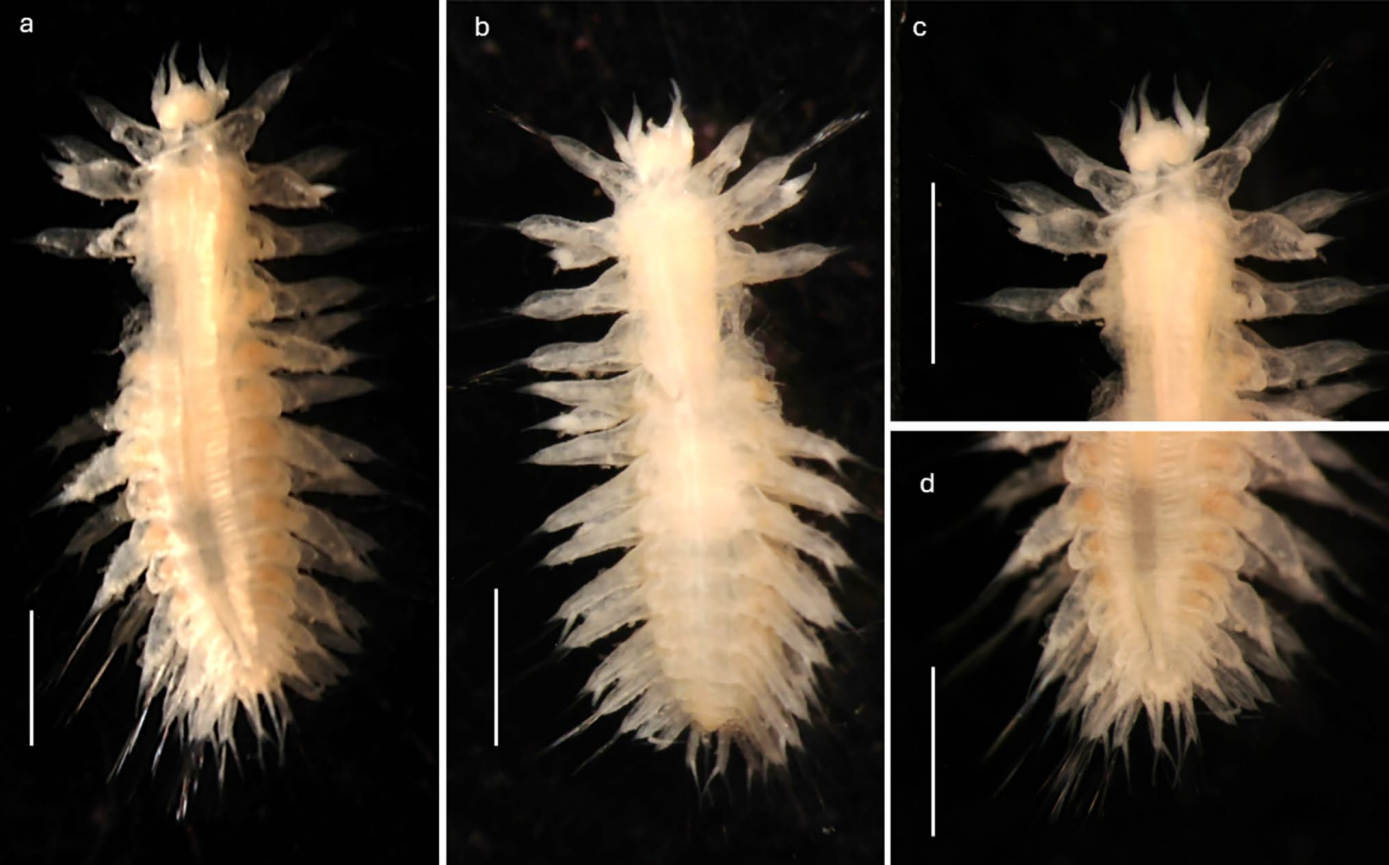
*Macellicephaloides veronikae* n. sp. Holotype (NHMUK. 2018.915). **a** Complete preserved specimen in dorsal view. **b** Complete preserved specimen in ventral view. **c** Anterior end in dorsal view. **d** Posterior end in dorsal view. All scale bars = 1000 µm

### Material examined

*Type material.* **Holotype** NHMUK. 2018.915: Amundsen Sea (Pine Island Bay), RRS James Clarke Ross 179, station BIO4-EBS-3F, collected on 07 March 2008, epibenthic sledge, supra-net, latitude −74.38964, longitude −104.7645, depth 523 m. **Paratype** NHMUK. 2018.916: Amundsen Sea (Pine Island Bay), RRS James Clarke Ross 179, station BIO4-EBS-3F, collected on 07 March 2008, epibenthic sledge, supra-net, latitude −74.38964, longitude −104.7645, depth 523 m. **Paratype** (SEM specimen) NHMUK. 2018.246: Amundsen Sea (Pine Island Bay), RRS James Clarke Ross 179, station BIO6-EBS-2B, collected on 12 March 2008, epibenthic sledge supra-net, latitude −71.17799, longitude −109.88426, depth 1005 m. **Other material:** NHMUK.2018.154, Amundsen Sea (Pine Island Bay), RRS James Clarke Ross 179, station BIO6-EBS-3C, collected on 12 March 2008, epibenthic sledge, epi-net, latitude −71.34783, longitude −110.01908, depth 482 m, 1 specimen; NHMUK. 2018.915-916, Amundsen Sea (Pine Island Bay), RRS James Clarke Ross 179, station BIO4-EBS-3F, collected on 07 March 2008, epibenthic sledge, supra-net, latitude −74.38964, longitude −104.7645, depth 523 m, 2 specimens; NHMUK.2018.434-435, Amundsen Sea (Pine Island Bay), RRS James Clarke Ross 179, station BIO5-EBS-3F, collected on 10 March 2008, epibenthic sledge, epi-net, latitude −73.98821, longitude −107.39726, depth 543 m, 2 specimens; NHMUK.2018.898-902, Amundsen Sea (Pine Island Bay), RRS James Clarke Ross 179, station BIO4-EBS-3F, collected on 07 March 2008, epibenthic sledge, epi-net, latitude −74.38964, longitude −104.7645, depth 523 m, 5 specimens; NHMUK. 2018.1069-1078, Amundsen Sea (Pine Island Bay), RRS James Clarke Ross 179, station BIO4-EBS-3C, collected on 07 March 2008, epibenthic sledge, epi-net, latitude −74.39848, longitude −104.63748, depth 505 m, 10 specimens.

#### Description (based on holotype and paratypes)

**Fig. 4:**
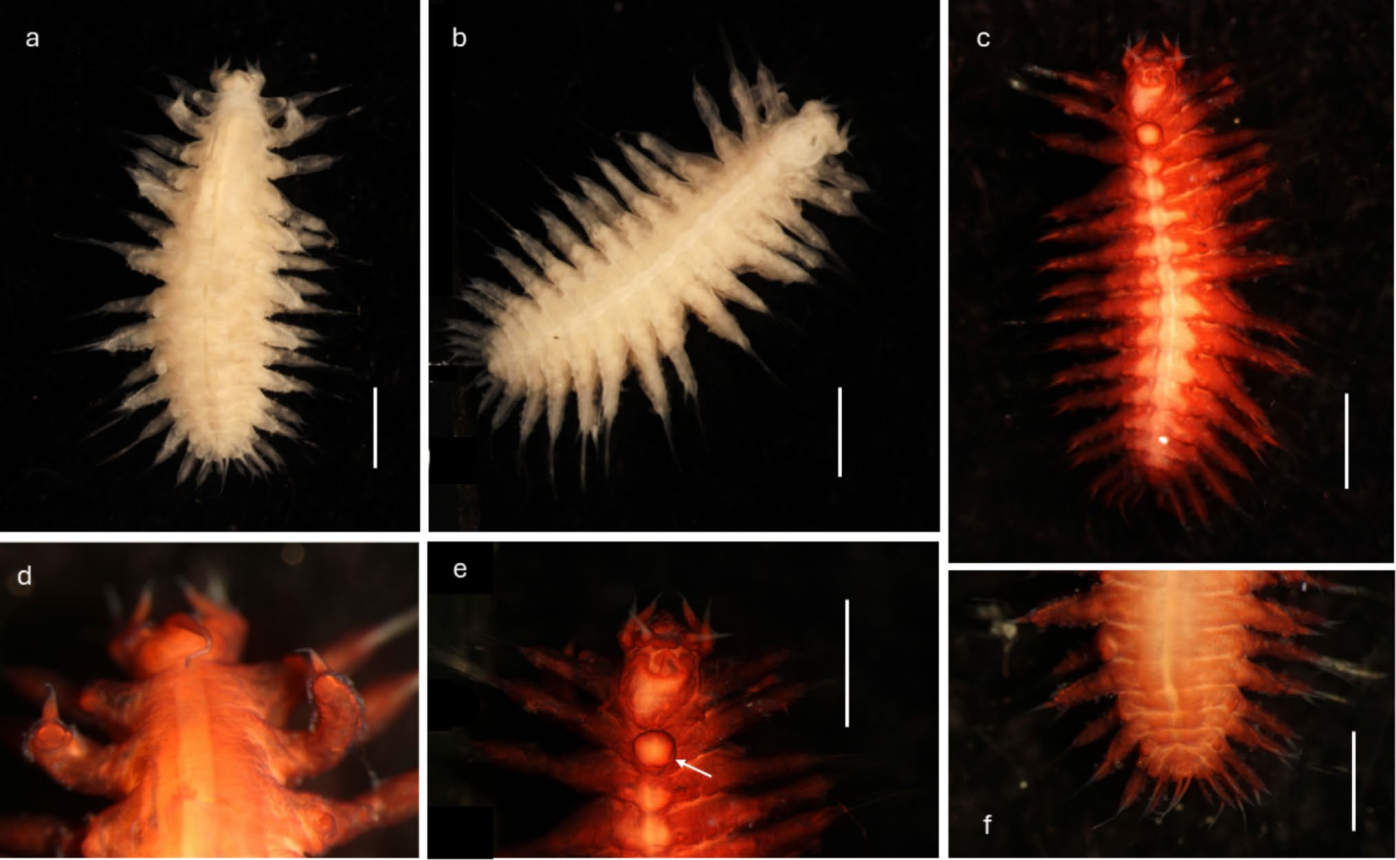
*Macellicephaloides veronikae* n. sp. Paratype (NHMUK. 2018.916). **a** Complete preserved specimen in dorsal view. **b** Complete preserved specimen in ventral view. **c** Complete preserved specimen in ventral view, specimen stained with Shirlastain A. **d** Anterior end in dorsal view, specimen stained with Shirlastain A.** e** Anterior end in ventral view, with ventral flap on segment 3 marked by arrow, specimen stained with Shirlastain A. **f** Posterior part of specimen in dorsal view. Scale bars = 1000 µm

**Fig. 5:**
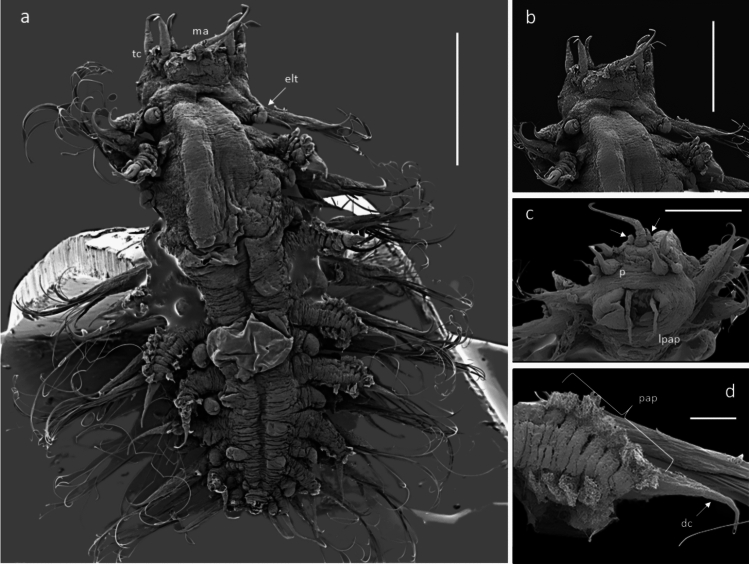
*Macellicephaloides veronikae* n. sp. paratype (NHMUK. 2018.246, SEM specimen), SEM micrograph. **a** Complete specimen in dorsal view (ma – median antenna, tc – tentacular cirri, elt – elytrophore). **b** Anterior end with prostomium in dorsal view. **c** Ventral view of the anterior end, showing partially everted proboscis with long pharyngeal papillae (lpap), palps (p), and papillae around the base of median tentaculophores (arrows). **d** Detail of dorsal cirrus with papillated cirrophores (pap) and smooth style (dc). Scale bars **a** = 1000 µm, **b** = 500 µm,** c** = 400 µm, **d = **100 µm

**Fig. 6:**
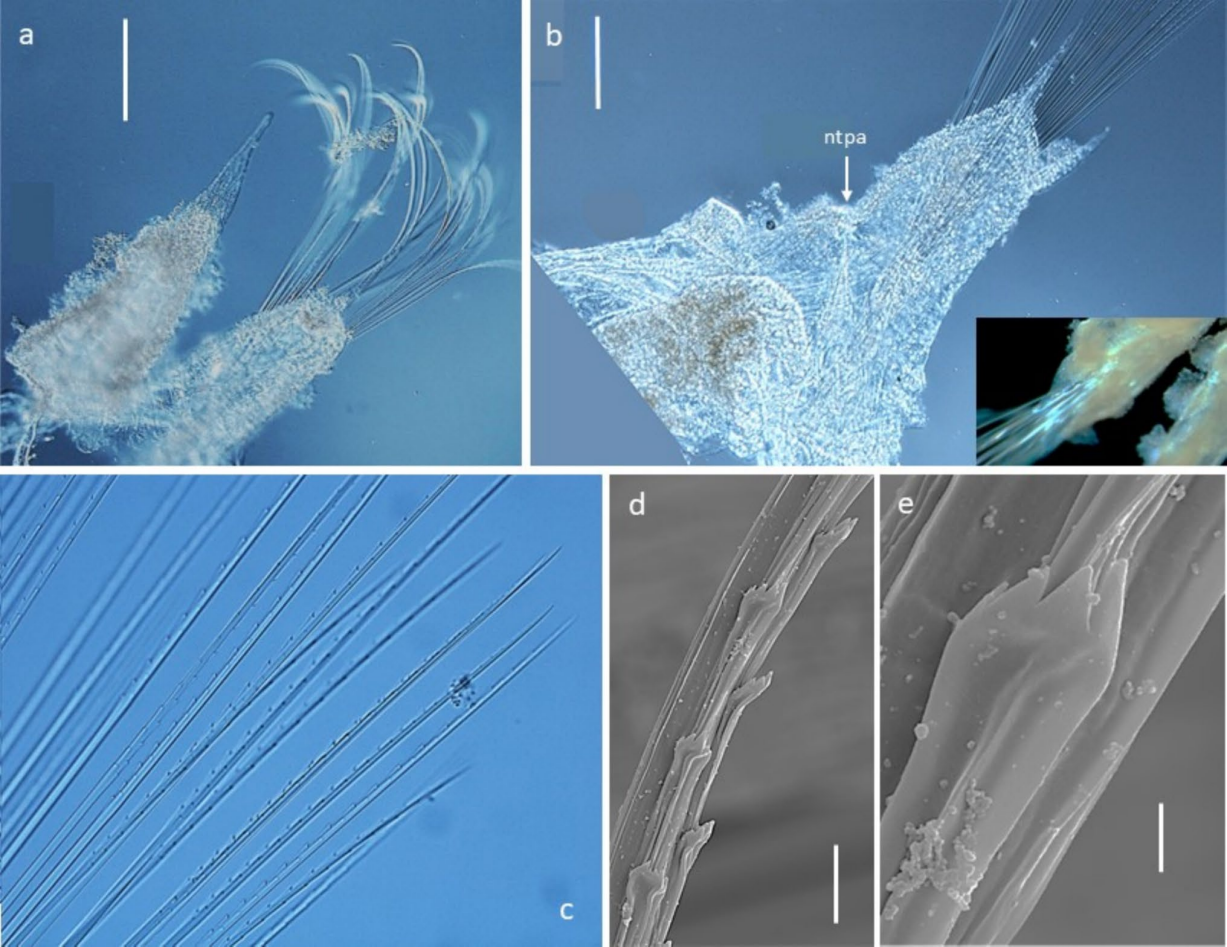
*Macellicephaloides veronikae* n. sp. paratype (NHMUK. 2018.246, SEM specimen) light microscopy in **a**–**c**, SEM micrograph **d**–**e**. **a** Cirrigerous parapodium. **b** Detail of elytragerous parapodium, notopodial acicula (ntpa) marked by arrow; insert showing papillae on neuropodium as seen using stereomicroscopy. **c** Fascicle of neurochaetae. **d** Arrangement of alternating spines on neurochaetal shaft. **e** Detail of forked neurochaetal spine with unequal teeth. Scale bars: **a**, **b** = 100 µm, **d** = 5 µm, **e** = 1 µm

Robust species up to 4.5 mm long with 16–19 segments (most specimens with 17 segments) (Figs. 3a, 4a) and 8 pairs of elytrophores (elytra missing); body integument smooth dorsally, but with papillae across ventrum arranged in five rows across each segment (Fig. 4c), on parapodia (Figs. 3b, 4a, 5a, d, 6b—insert, 7j) and around ceratophores of median antenna and tentacular cirri (Fig. 5b, c). Holotype NHMUK. 2018.915 complete (Fig. 3a–d), 4.1 mm long, 1.8 mm wide (including parapodia), and 0.9 mm wide (excluding parapodia) for 17 segments (including tentacular segment). Body robust, compact, dorsoventrally flattened; colour in alcohol pale yellow (Figs. 3a, b). Paratype NHMUK. 2018.916 complete (Fig. 4a–f), 4.2 mm long, 2.5 mm wide (including parapodia), and 1.1 mm wide (excluding parapodia) for 18 segments (including tentacular segment). Paratype (SEM specimen) NHMUK. 2018.246 (Fig. 5a–d), 3.5 mm long, 1.9 mm wide (including parapodia), and 0.8 mm wide (excluding parapodia) for 17 segments (including tentacular segment).

**Fig. 7:**
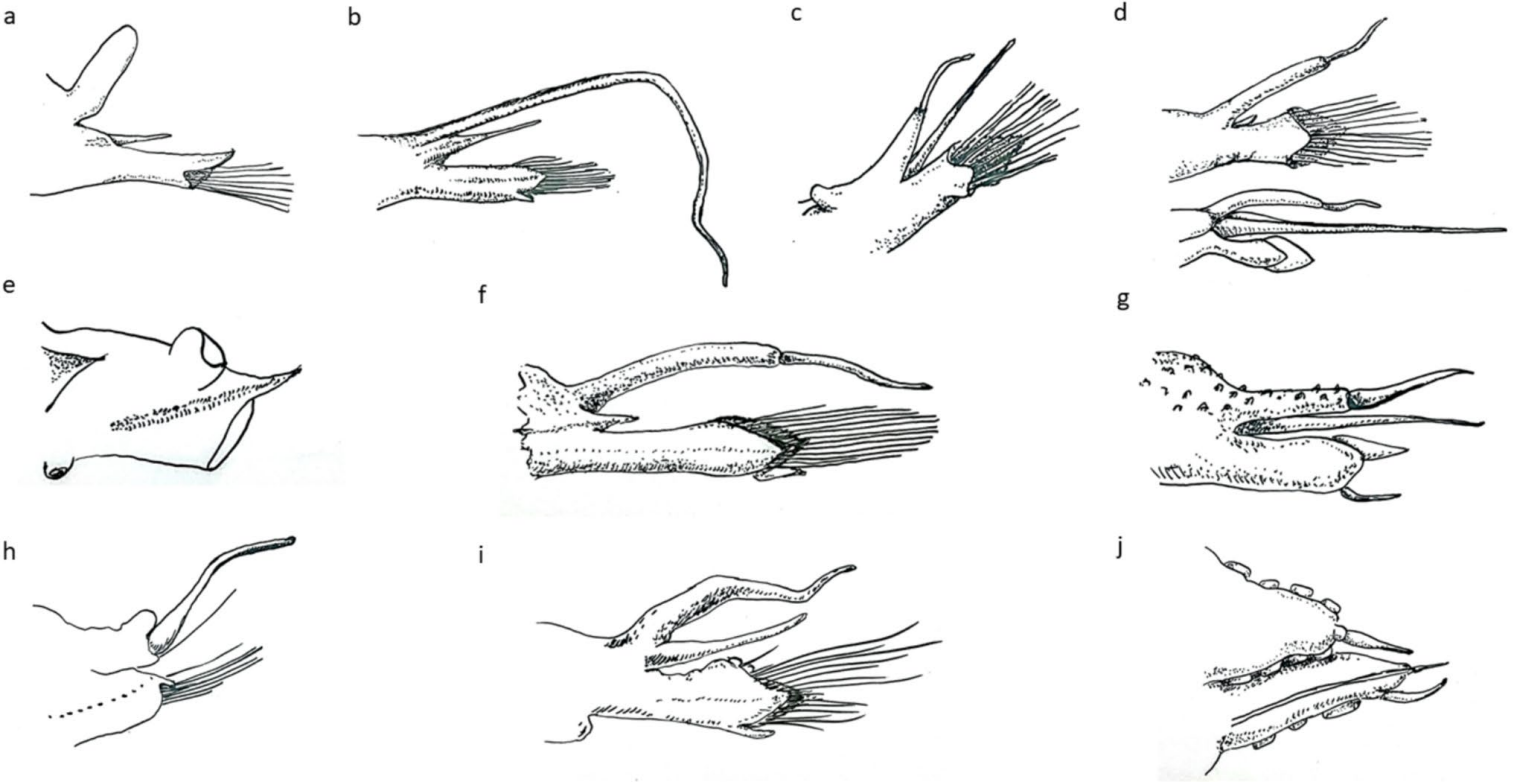
Comparison of shapes of parapodia of all known species of *Macellicephaloides* (**a-i**) and *M. veronikae* sp. n. (**j**), all parapodia cirrigerous with the exception of (**e**), line drawings not drawn to scale. **a** *M*. *alvini* after Pettibone (1989a, b), style of dorsal cirrus missing. **b** *M. grandicirra* after Uschakov (1955).** c** *M. verrucosa* after Uschakov (1955). **d** Anterior parapodium of *M. vitiazi* with reduced notopodium (top), posterior parpodium of *M. vitiazi* with elongated notopodium (bottom), both after Uschakov (1955). **e** elytragerous parapodium *M. improvisa* after Levenstein (1982). **f** *M. sandwichensis* after Levenstein (1975). **g** *M. villosa* after Levenstein (1982) showing papillae. **h** *M. moustachu* after Bonifácio and Menot (2019).** i** *M. uschakovi* after Levenstein (1971). **j** *M*. *veronikae* sp. n., cirrigerous parapodium showing papillae bordering cirrophore and neuropodium

Prostomium bilobed, about as wide as long, with shallow median notch (Fig. 5b). Eyes absent. Median antenna (Fig. 5a–c) with large ceratophore, tufts of papillae laterally at the base of the ceratophore (Fig. 5c), style of antenna moderately long tapering filament, reaching to 2nd segment (longest of all prostomial appendages); lateral antennae absent; frontal filaments absent. Palps smooth, short and robust, wide and thick basally, tapering distally (Fig. 5a–c). Pharynx partially everted in both paratypes; one pair of very long lateral papillae was observed (Fig. 5c). Jaws not observed.

Tentacular cirri similar in size and form to palps, consisting of bulbous tentaculophores and short, smooth distally tapering styles; the bases of tentaculophores with tufts of papillae. Second segment with elytrophores, subbiramous parapodia, with chaetae and ventral cirri. On ventrum of segment 3, a small broadly rounded fleshy pad present (Fig. 4e).

Body always with 8 pairs of elytrophores on segments 2, 4, 5, 7, 9, 11, 13, and 15. Elytrophores inflated, knob-shaped (Fig. 5a). Elytra missing.

Parapodia subbiramous. Notopodia extremely reduced, with acicula (Fig. 6a, b). Neuropodia elongated, distally narrowly pointed, with acicular lobe (Figs. 6b, 7j); neuropodia dorsally and ventrally bordered by papillae (Figs. 3b, 4b, 6b-insert, 7j). Cirrigerous segments with massive, somewhat inflated cirrophores bordered by papillae (Figs. 4a, d, 5a, d, 7j); styles of dorsal cirri similar to the tentacular cirri and palps, somewhat flask-shaped (Figs. 4d, 5d, 6a, 7j) in the anterior part of the body, becoming slightly longer posteriorly. Dorsal tubercules not developed. Ventral cirri present from segment 2 to last segment; inserted basally on segment 2, where very short; in subsequent segments attached subdistally on neuropodium, short, cirriform, tapering, becoming longer posteriorly (Fig. 7j).

Notochaetae absent. Neurochaetae numerous, very long, slender capillaries (Fig. 6a) with alternating rose-bush like spines, each spine forked distally with 4–5 sharp unequal teeth (Fig. 6d–e). Nephridial papillae enlarged on ventrum of chaetigers 6–15, observed as raised bulges. Pygidium tubular, enclosed by the posteriormost segments, with two long thin, anal cirri (Figs. 3d, 4f).

#### DNA

16S and 18S genes were successfully sequenced for this species, GenBank accession numbers KX867331-KX867344 (Brasier et al. 2016) and PV911683-PV911684 for holotype NHMUK. 2018.915 (this study).

#### Distribution

Southern Ocean, Amundsen Sea, including deep-shelf troughs of Pine Island Bay, in depths of ~ 500– ~ 1000 m.

#### Etymology

This species is dedicated to Dr. Veronika Lukášová (https://www.veronikalukasova.com), artist, author, natural history enthusiast, and collector and to her incredible life.

#### Remarks

This is the tenth species to be described in the genus *Macellicephaloides*. The species has been assigned to this genus based on the combination of the following characters (see Bonifácio and Menot 2019): body with < 21 segments, 8 pairs of elytra, bilobed prostomium with median antenna, lateral antennae absent, parapodia with reduced notopodia, and well-developed neuropodia, with neurochaetae only.

*Macellicephaloides veronikae* sp. n. can be easily distinguished from all other known species by the presence of papillae, attached to the bases of ceratophores of median antenna and tentacular cirri, but most prominently observed on neuropodia and cirrophores of dorsal cirri, and ventrum, where arranged in five rows across each segment (Fig. 4c). Of the known species, only *M. villosa* has the body surface papillated, but the papillae are restricted to the dorsum and cirrophores of dorsal cirri (Fig. 7g), while all other species have a smooth integument (see comparative Fig. 7a–f, h, i).

With the exception of *M. villosa*, the forked nature of neurochaetal spines was never specifically mentioned in the previous descriptions, although it was depicted in SEM image of *M. moustachu*, (Fig. 16 J in Bonifácio and Menot 2019).While this structure of neurochaetae is best observed using SEM (Fig. 6d–e), this character can be approximated even under high level (oil) magnification using light microscopy [Neal pers. observation; Levenstein (1971, Fig. 2b)] and it is possible that the forking of spines is present in other *Macellicephaloides* species, but has previously been overlooked.

The availability of molecular data for *M. veronikae* sp. n. shows that number of segments is not fixed in this species, similar to the finding of Bonifácio and Menot (2019) for *M. moustachu*. Specimens belonging to *M. veronikae* sp. n. as determined by 16S marker (this study, Brasier et al. 2016) possess 16–19 segments, with most having 17 segments. Brasier et al. (2016) reported K2P distances between *Macellicephaloides* sp. (formalized here as *M. veronikae* n. sp.) sequences ranged from 0.00 to 3.56%, failing to find the barcoding gap in the absence of data from COI marker. Except for *M. moustachu,* all previously known species were described based on morphology only, often from a single specimen, and thus intraspecific variation in number of segments is unknown. A tabulated key to all known species of *Macellicephaloides* is provided below (Table 2).

**Table 2:** Comparison of selected morphological characters among all known species of *Macellicephaloides*

	Type locality	Type depth (m)	No. of specimens examined	No. of segments	Integument	Development of notopodium (see Fig. 7)	Cirrophore of dorsal cirrus (see Fig. 7)	Neurochaetae
*Macellicephaloides alvini* Pettibone, 1989a, b	Gulf of California, Eastern Pacific	2004	1	17	smooth	~ 1/2 the length of neuropodium	quite robust, cylindrical, slightly shorter then neuropodium	slender, with two rows of delicate spines
*Macellicephaloides grandicirra* Uschakov, 1955	Kuril-Kamchatka Trench, NW Pacific	8100–9950	43	17	smooth	the same length as notopodium	extremely thin and long, 3–5 × the length of neuropodium	slender, with two rows of spines
*Macellicephaloides improvisa* Levenstein, 1982	Kuril-Kamchatka Trench, NW Pacific	?	1	18	smooth	very reduced, much shorter than neuropodium	very short	unknown
*Macellicephaloides moustachu* Bonifácio & Menot, 2018	CCZ, eastern Pacific	4398	10	14–16 (11 in juvenile)	smooth	very reduced, much shorter than neuropodium	large, cylindrical, elongate, with pointed projection	slender, distally with spines along both margins, with pointed tips [forked spines imaged but not described in Bonifacio and Menot (2019)]
*Macellicephaloides sandvichensis* Levenstein, 1975	South Sandwich Trench, South Atlantic	7200–7934	?	17	smooth	small, much shorter than neuropodium	very slender, about the length of neuropodium	slender, with two rows of spines, distally with slender tooth (hook)
*Macellicephaloides uschakovi* Levenstein, 1971	Kuril-Kamchatka Trench, NW Pacific	8120	16	20–21	smooth	the same length as notopodium	somewhat thickened, ~ 1/2 length of neuropodia	with two rows of distinct spines, distally gently curved
*Macellicephaloides verrucosa* Uschakov, 1955	Kuril-Kamchatka Trench, NW Pacific	7210–7230	3	16	smooth	slightly longer then neuropodium	thick, slightly shorter then neuropodium	slender, with two rows of delicate spines
*Macellicephaloides villosa* Levenstein, 1982	Japan Trench, NW Pacific	7370–7380	1	21	papillated—dorsum, parapodia	very long, longer then neuropodium	slender, elongated, slightly short then neuropodium, papillated	slender, distally with spines along both margins; spines forked with multiple teeth
*Macellicephaloides vitiazi* Uschakov, 1955	Kuril-Kamchatka Trench, NW Pacific	7210–8430	2	16	smooth	variable along the length of the body, extremely short in the anterior in mid segments, very long in the posterior segments	very slender, about the length of neuropodium	slender, with two rows of delicate spines
*Macellicephaloides veronikae*** n. sp.**	Amundsen Sea, Southern Ocean	~ 500–1000	27 (15*)	16–19	papillated—ventrum, parapodia	very reduced, much shorter than neuropodium	massive, somewhat inflated cirrophores bearing papillae on the edges	slender, distally with spines along both margins; spines forked into ~ 4 unequal teeth

^*^Specimens used in an unpublished stable isotope analysis study, now represented by DNA extractions only with the associated sequences published by Brasier et al. (2016)

*Macellicephaloides veronikae* sp. n. is the third species of its genus to be described from a non-trench environment and represents the shallowest known record of this genus to date. While an evolutionary link between fauna of the deep-sea and polar shelves has been previously proposed (e. g. Strugnell et al. 2011), the complex bathymetry of the Pine Island Bay in the Amundsen Sea, with its deep (up to 1500 m) troughs carved by glaciers, is likely to harbour further taxa with deep-sea relatives (Riehl and Kaiser 2012; Neal et al. 2017, 2018). The Antarctic shelf can be considered a deep-water analogue, owing to the cold temperatures, darkness (even if seasonal) and greater depth of the shelf due to ice-loading. More species are likely to be discovered with further sampling effort from Antarctic deep-sea realms that can provide further insights into the biodiversity and evolutionary history of the region.

## Data Availability

Sequence data that support the findings of this study have been deposited in GenBank. Specimens were deposited at the Natural History Museum London.
